# Effect of low body mass index on clinical recovery after fusion surgery for osteoporotic vertebral fracture: A retrospective, multicenter study of 237 cases

**DOI:** 10.1097/MD.0000000000032330

**Published:** 2022-12-30

**Authors:** Gen Inoue, Masayuki Miyagi, Wataru Saito, Eiki Shirasawa, Kentaro Uchida, Naobumi Hosogane, Kei Watanabe, Keiichi Katsumi, Takashi Kaito, Tomoya Yamashita, Hiroyasu Fujiwara, Yukitaka Nagamoto, Kenya Nojiri, Satoshi Suzuki, Eijiro Okada, Seiji Ueda, Tomohiro Hikata, Yuta Shiono, Kota Watanabe, Hidetomi Terai, Koji Tamai, Yuji Matsuoka, Hidekazu Suzuki, Hirosuke Nishimura, Atsushi Tagami, Shuta Yamada, Shinji Adachi, Seiji Ohtori, Takeo Furuya, Sumihisa Orita, Kazuhide Inage, Toshitaka Yoshii, Shuta Ushio, Haruki Funao, Norihiro Isogai, Katsumi Harimaya, Seiji Okada, Kenichi Kawaguchi, Nobuhiko Yokoyama, Hidekazu Oishi, Toshio Doi, Katsuhito Kiyasu, Shiro Imagama, Kei Ando, Kazuyoshi Kobayashi, Daisuke Sakai, Masahiro Tanaka, Atsushi Kimura, Hirokazu Inoue, Atsushi Nakano, Shota Ikegami, Masayuki Shimizu, Toshimasa Futatsugi, Kenichiro Kakutani, Takashi Yurube, Kazuyoshi Nakanishi, Masashi Oshima, Hiroshi Uei, Yasuchika Aoki, Masahiko Takahata, Akira Iwata, Hirooki Endo, Shoji Seki, Hideki Murakami, Satoshi Kato, Katsuhito Yoshioka, Michio Hongo, Tetsuya Abe, Toshinori Tsukanishi, Masashi Takaso, Ken Ishii

**Affiliations:** a Department of Orthopaedic Surgery, Kitasato University, Sagamihara City, Kanagawa, Japan; b Shonan University of Medical Sciences Research Institute, Chigasaki City, Kanagawa, Japan; c Department of Orthopaedic Surgery, Kyorin University, Mitaka City, Tokyo, Japan; d Department of Orthopaedic Surgery, Niigata University, Chuo-ku, Niigata City, Japan; e Department of Orthopaedic Surgery, Osaka University, Suita City, Osaka, Japan; f Department of Orthopaedic Surgery, Keio University, Shinjuku-ku, Tokyo, Japan; g Department of Orthopaedic Surgery, Spine Center, Kitasato Institute Hospital, Minato-ku, Tokyo, Japan; h Department of Orthopaedic Surgery, Osaka City University, Abeno-ku, Osaka City, Japan; i Department of Orthopaedic Surgery, Tokyo Medical University, Shinjuku-ku, Tokyo, Japan; j Department of Orthopaedic Surgery, Nagasaki University, Nagasaki City, Japan; k Department of Orthopaedic Surgery, Chiba University, Chuo-ku, Chiba City, Japan; l Center for Medical Engineering, Chiba University, Inage-ku, Chiba City, Japan; m Department of Orthopaedic Surgery, Tokyo Medical and Dental University, Bunkyo-ku, Tokyo, Japan; n Department of Orthopaedic Surgery, International University of Health and Welfare, Narita City, Chiba, Japan; o Spine and Spinal cord Center, International University of Health and Welfare Mita Hospital, Minato-ku, Tokyo, Japan; p Department of Orthopaedic Surgery, Kyushu University, Higashi-ku, Fukuoka City, Japan; q Department of Orthopaedic Surgery, Kochi University, Oko-cho Kohasu, Nankoku City, Kochi, Japan; r Department of Orthopaedic Surgery, Nagoya University, Showa-ku, Nagoya City, Aichi, Japan; s Department of Orthopaedic Surgery, Tokai University, Isehara City, Kanagawa, Japan; t Department of Orthopaedic Surgery, Jichi Medical University, Shimotsuke City, Tochigi, Japan; u Department of Orthopaedic Surgery, Osaka Medical College, Takatsuki City, Osaka, Japan; v Department of Orthopaedic Surgery, Shinshu University, Matsumoto City, Nagano, Japan; w Department of Orthopaedic Surgery, Kobe University, Chuou-ku, Kobe City, Hyogo, Japan; x Department of Orthopaedic Surgery, Nihon University Itabashi Hospital, Itabashi-ku, Tokyo, Japan; y Department of Orthopaedic Surgery, Eastern Chiba Medical Center, Togane City, Chiba, Japan; z Department of Orthopaedic Surgery, Hokkaido University, Kita-ku, Sapporo City, Hokkaido, Japan; aa Department of Orthopaedic Surgery, Iwate Medical University, Yahaba-cho, Iwate, Japan; bb Department of Orthopaedic Surgery, University of Toyama, Toyama City, Japan; cc Department of Orthopaedic Surgery, Nagoya City University, Mizuho-ku, Nagoya City, Aichi, Japan; dd Department of Orthopaedic Surgery, Kanazawa University, Kanazawa City, Japan; ee Department of Orthopaedic Surgery, Akita University, Akita City, Japan; ff Department of Orthopaedic Surgery, University of Tsukuba, Tsukuba City, Ibaraki, Japan.

**Keywords:** body mass index, fusion surgery, osteoporosis, vertebral fracture

## Abstract

A retrospective multicenter study. Body mass index (BMI) is recognized as an important determinant of osteoporosis and spinal postoperative outcomes; however, the specific impact of BMI on surgery for osteoporotic vertebral fractures (OVFs) remains inconclusive. This retrospective multicenter study investigated the impact of BMI on clinical outcomes following fusion surgery for OVFs. 237 OVF patients (mean age, 74.3 years; 48 men and 189 women) with neurological symptoms who underwent spinal fusion were included in this study. Patients were grouped by World Health Organization BMI categories: low BMI (<18.5 kg/m^2^), normal BMI (≥18.5 and <25 kg/m^2^), and high BMI (≥25 kg/m^2^). Patients’ backgrounds, surgical method, radiological findings, pain measurements, activities of daily living (ADL), and postoperative complications were compared after a mean follow-up period of 4 years. As results, the proportion of patients able to walk independently was significantly smaller in the low BMI group (75.0%) compared with the normal BMI group (89.9%; *P* = .01) and the high BMI group (94.3%; *P* = .04). Improvement in the visual analogue scale for leg pain was significantly less in the low BMI group than the high BMI group (26.7 vs 42.8 mm; *P* = .046). Radiological evaluation, the Frankel classification, and postoperative complications were not significantly different among all 3 groups. Improvement of pain intensity and ADL in the high BMI group was equivalent or non-significantly better for some outcome measures compared with the normal BMI group. Leg pain and independent walking ability after fusion surgery for patients with OVFs improved less in the low versus the high BMI group. Surgeons may want to carefully evaluate at risk low BMI patients before fusion surgery for OVF because poor clinical results may occur.

## 1. Introduction

Globally, osteoporosis is a major medical problem, and of particular concern in Japan, a country with a largest aging population in the world. In a Japanese population-based cohort study, the reported prevalence of osteoporosis in the lumbar spine was 3.4% in men and 19.2% in women, with a tendency to be higher with advanced age in both sexes, but particularly in females.^[1]^ Worldwide, vertebral fractures are the most common osteoporosis-related fractures, and 2 Japanese longitudinal cohort studies report a higher incidence of vertebral fractures in older women than in men of the same age group, suggesting osteoporosis could be influenced by menopause.^[2,3]^ Evidence shows low bone mineral density (BMD) is consistently correlated with an increased risk of osteoporotic vertebral fracture (OVF) and low body weight. Body mass index (BMI), measured by body weight in kg/height in m^2^, can indicate whether one is underweight, normal weight, or overweight based on height. Low BMI ≤ 20 kg/m^2^ is recognized as 1 risk factor for osteoporosis with the probability of increasing the incidence of osteoporosis-related fractures including OVFs.^[4,5]^ Several studies report a positive correlation between BMI and BMD, with an 8% to 12% decrease in the risk of osteoporosis with a BMI increase of 1 kg/m^2^.^[5–8]^ Reportedly, BMI is also inversely correlated with postmenopausal bone loss.^[9]^ In contrast, higher estrogen levels in moderately obese women have protected them against bone loss compared with woman of normal weight, despite obesity increasing the risk of adverse health outcomes including hypertension, dyslipidemia, coronary heart disease, stroke, type 2 diabetes mellitus, gallbladder disease, sleep apnea, osteoarthritis, cancer, mental illness, and mortality.^[9,10]^ A higher complication rate following spinal surgery in obese patients with a BMI ≥ 30 kg/m^2^ has been reported.^[11]^ Based on background data of this nature, we hypothesized that BMI is an important determinant of OVF surgical results, particularly in older adults who frequently undergo operations of this nature.

We conducted a retrospective, multicenter study to measure the impact of BMI on OVF surgery. The study examines patients’ background data, radiological and clinical outcomes including pain and ADL evaluations, and complications.

## 2. Materials and Methods

### 2.1. Patient selection

This was a retrospective, multicenter study conducted through the Japan Association of Spine Surgeons with Ambition comprising institutions across Japan. The study was approved by the institutional review boards of all participating institutions. A total of 237 patients (mean age: 74.3 ± 8.1; male: 48; female: 189) were included from 2005 to 2014. Eligible patients met the following inclusion criteria: OVF with vertebral collapse or nonunion at T1 to L3; existence of neurological deficits, including motor weakness and/or sensory impairment including pain; underwent fusion surgery with instrumentation; minimum follow-up of 1 year; and availability of pre-operative height and weight data. Indications for, and methods of surgery were decided by each doctor individually and were not standardized among the institutes. Exclusion criteria were: patients with collagen diseases, Parkinson’s disease, malignant tumor, or depression; patients prescribed ≥ 10 mg/day of steroids; patients who underwent vertebroplasty or balloon kyphoplasty alone without fusion surgery; and patients lacking information about either their height or weight before surgery. Informed consent was obtained at each institute. A datasheet was sent to each spine surgeon, who populated it with information about their patients’ backgrounds and relevant clinical or radiological data.

### 2.2. Data collection

Patients’ demographics were collected retrospectively. Background data included age, sex, height, weight, BMI, BMD in the lumbar spine and the femoral neck, comorbidities of diabetes mellitus, cerebrovascular disease, renal dysfunction, probable cause of OVF, number of prevalent vertebral fractures before surgery, preoperative pharmacological treatment for osteoporosis (e.g., bisphosphonates, teriparatide, selective estrogen receptor modulators, and/or others), current use of steroid use.

The surgical information collected incorporated days from knowledge of the fracture to surgery, operative time, estimated blood loss, level of OVF targeted surgically, surgery by stage (1- or 2-stages), surgical approach (anterior/lateral, posterior, and/or combined), number of fused vertebral levels, local kyphotic angle (preoperatively, postoperatively, and at final follow-up), corrective angle during surgery, and the reduction of local kyphosis after surgery.

The assessment of outcome measures for activities of daily living (ADL) between preoperative and final follow-ups included the Japanese Orthopaedic Association (JOA) score for lumbar disease (a full score was 15 points and comprised: subjective symptoms = 9 points, clinical signs = 6 points, and bladder and bowel dysfunction=[−6 points]), visual analogue scale (VAS) for low back pain (LBP) and leg pain, walking ability (grade 1 = unable to walk, grade 2 = requires caregiver assistance, grade 3 = ambulates using a walker or 2 canes, grade 4 = walks using 1 cane, and grade 5 = walks unaided), and Frankel classification (data obtained before surgery and at final follow-up).

Complication monitoring included the collection of perioperative complication rates that required treatment and occurred within 6 weeks postoperatively (i.e., delirium, dural tear, surgical site infection, hematoma, pneumonia, and/or deep vein thrombosis). The incidence of mechanical failure was tracked until final follow-up and involved screw loosening and backout, postoperative vertebral fracture, and/or nonunion of fractured vertebra, etcetera, as well as single or multiple re-operation rates.

### 2.3. Assessments

Among the 237 patients included in this study, the mean postoperative follow-up period was 4.0 years. Patients were divided into 3 groups based on BMI categories defined by the World Health Organization as follows: low BMI (n = 24 < 18.5 kg/m^2^), normal BMI (n = 160, ≥18.5−<25 kg/m^2^), and high BMI (n = 53, ≥25 kg/m^2^). All variables collected in this study were compared among the 3 groups and the impact of BMI on OVFs treated using fusion surgery was evaluated.

### 2.4. Statistics

Statistical analysis was carried out using IBM SPSS software version 26.0 (Chicago, IL). One-way ANOVA was used to compare differences of the mean values of continuous variables and Pearson’s chi-squared test was used to compare categorical variables among the 3 groups. When significant differences among the 3 groups were observed, post hoc pairwise comparisons were performed using the Tukey procedure, Games-Howell test, or Pearson’s chi-squared tests. *P* values < .05 were considered statistically significant. Because patients occasionally filled out questionnaires incompletely, the exact number of patients for each specific analysis varied slightly.

## 3. Results

### 3.1. Patient background data

The background data of patients is summarized by BMI category and overall (Table 1). The overall (N = 237) mean BMI was 22.8 ± 3.9 kg/m^2^. A probable cause of OVFs was identified in 79.2% of those patients, whose fractures were predominantly in the thoracolumbar region (T11 − L2: 93.7%) followed by the middle thoracic level (T6 − 10: 6.3%). No patients had upper thoracic region (T1 − 5) OVFs. Age, weight, and BMI were statistically different among the 3 BMI groups. post hoc comparisons revealed that the high BMI group was significantly younger than the normal BMI group (71.6 ± 7.9 vs 75.3 ± 7.9). Weight and BMI were significantly different among the 3 groups, but not height. Sex, BMD, comorbidities, probable cause of OVF, number of prevalent vertebral fractures before injury, osteoporosis treatment before surgery, pharmacological treatment for osteoporosis, recent history of steroid use ≥ 10 mg, and level of surgical OVF showed no significant differences.

**Table 1:** Patients’ background.

	BMI				
	Low, n = 24	Normal, n = 160	High, n = 53	All, n = 237	*P* value
Age, yrs	72.8 ± 8.1	75.3 ± 7.9‡	71.6 ± 7.9‡	74.3 ± 8.1	**.01**
Male/female sex, n	3/ 21	33/ 127	12/ 41	48/189	.58
Height, cm	154.9 ± 8.7	152.3 ± 7.7	150.4 ± 8.5	152.1 ± 8.0	.07
Weight, kg	40.3 ± 5.5*†	50.8 ± 6.3*‡	63.8 ± 8.4†‡	52.7 ± 9.5	**<.01**
BMI, kg/m^2^	16.7 ± 1.2*†	21.8 ± 1.7*‡	28.2 ± 3.1†‡	22.8 ± 3.9	**<.01**
BMD, g/cm^2^					
Lumbar spine	0.73 ± 0.17	0.68 ± 0.12	0.80 ± 0.16	0.72 ± 0.14	.10
Femoral neck	0.56 ± 0.16	0.60 ± 0.15	0.68 ± 0.19	0.62 ± 0.16	.83
Comorbidity, n (%)					
Diabetes mellitus	7 (29.2)	38 (23.8)	14 (26.4)	59 (24.9)	.81
Cardiovascular disease	3 (12.5)	37 (23.1)	15 (28.3)	55 (23.2)	.31
Renal dysfunction	3 (12.5)	12 (7.5)	3 (5.7)	18 (7.6)	.58
Probable cause of OVF, n (%)	16 (80.0)	120 (79.5)	35 (77.8)	171 (79.2)	.97
No. of prevalent vertebral fractures before injury	0.63 ± 0.82	0.61 ± 1.08	0.68 ± 1.16	0.63 ± 1.07	.82
Osteoporosis treatment before surgery, n (%)	9 (37.5)	57 (35.6)	18 (34.0)	84 (35.4)	.95
Pharmacological treatment for osteoporosis, n (%)					
Bisphosphonate	7 (29.2)	33 (20.6)	15 (28.3)	55 (23.2)	.40
Teriparatide	2 (8.3)	10 (6.3)	0 (0.0)	12 (5.1)	.15
SERM	0	5 (3.1)	1 (1.9)	6 (2.5)	.63
Other	0	11 (6.9)	3 (5.7)	14 (5.9)	.41
Current use of steroid ≥ 10 mg, n (%)	1 (4.2)	3 (1.9)	2 (3.8)	6 (2.5)	.65
Level of surgical OVF, n (%)					
T1–5	0	0	0	0	.66
T6–10	2 (8.3)	11 (6.9)	2 (3.8)	15 (6.3)	
T11–L2	22 (91.7)	149 (93.1)	51 (96.2)	222 (93.7)	

Values represent mean ± standard deviation or n (%).

BMD = bone mineral density, BMI = body mass index, OVF = osteoporotic vertebral fracture, SERM = selective estrogen receptor modulator.

Comparison among 3 groups were performed using 1-way ANOVA for continuous variables and Pearson’s χ^2^ test for categorical variables. A *P* value < .05 was statistically significant. post hoc pairwise comparisons between the low and the normal BMI groups:

* *P* < .05. Comparison between the low and the high BMI groups:

† *P* < .05. Comparison between the normal and the high BMI groups:

‡ *P* < .05.

### 3.2. Surgical method and radiological evaluation

Factors related to surgery are shown in Table 2. The mean duration between fracture and surgery was 235 days. Operative time and estimated blood loss were not significantly different among the groups, and the overall mean values were 237.0 minutes and 666.1 mL, respectively. Most surgeries (97.2%) were performed in 1 stage and the posterior approach alone was used for 86.1% of all cases. Local kyphosis decreased from a preoperative 25.8^o^ to a postoperative 7.7°. Overall averages showed the number of fused vertebral levels was 4.08, the corrective angle during surgery was 18.1°, and the loss of correction angle after surgery was 7.0° and not statistically different among the groups 4.0 years after surgery. All factors related to surgical method and radiological evaluation during surgery were not significantly different among the 3 groups.

**Table 2:** Factors related to surgery.

	BMI				
	Low, n = 24	Normal, n = 160	High, n = 53	All, b = 237	*P* value
Duration between fracture and surgery, d	122.4 ± 83.3	242.7 ± 316.6	256.4 ± 308.7	235.9 ± 302.2	.46
Postoperative follow-up period, d	1437.5 ± 695.4	1451.7 ± 582.6	1476.6 ± 616.0	1455.8 ± 599.6	.96
Surgical time, min	227.8 ± 73.5	241.6 ± 96.6	228.0 ± 81.2	237.0 ± 90.9	.59
EBL, mL	498.4 ± 392.2	760.0 ± 1612.9	474.8 ± 435.5	666.1 ± 1339.5	.36
Surgery by stage, n (%)					
One stage	20 (100.0)	146 (96.7)	46 (97.9)	212 (97.2)	.67
Two stages	0	5 (3.3)	1 (2.1)	6 (2.8)	
Approach, n (%)					
Anterior or lateral	0	9 (5.6)	5 (9.4)	14 (5.9)	.24
Posterior	24 (100.0)	136 (85.0)	44 (83.0)	204 (86.1)	
Combined	0	15 (9.4)	4 (7.5)	19 (8.0)	
Number of fused levels	4.79 ± 2.13	3.98 ± 1.38	4.02 ± 1.92	4.08 ± 1.62	.07
Local kyphosis angle, °					
Before surgery	31.5 ± 17.5	25.0 ± 13.1	25.8 ± 13.2	25.8 ± 13.7	.09
After surgery	7.5 ± 11.1	6.8 ± 10.3	10.6 ± 12.1	7.7 ± 10.8	.09
Final follow up	13.1 ± 10.7	14.5 ± 12.7	16.4 ± 12.4	14.8 ± 12.4	.51
Corrective angle during surgery, °	24.0 ± 19.0	18.2 ± 12.0	15.2 ± 12.2	18.1 ± 13.1	.09
Correction angle loss after surgery, °	5.6 ± 7.1	7.7 ± 8.1	5.8 ± 6.0	7.0 ± 7.6	.17

Values represent mean ± standard deviation or n (%).

BMI = body mass index, EBL = estimated blood loss.

Comparison among 3 groups were performed using 1-way ANOVA for continuous variables and Pearson’s χ^2^ test for categorical variables. A *P* value < .05 was statistically significant.

### 3.3. Pain intensity and ADL level

**Figure 1.**
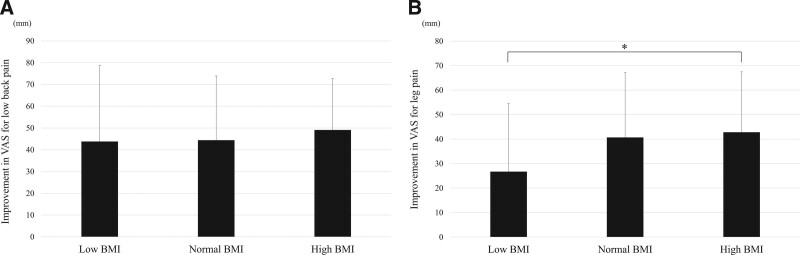
Visual analogue scale score improvements for low back pain (A) and leg pain (B). Values are mean ± standard deviation; *P < .05 in post hoc pairwise comparisons with Tukey procedure after a 1-way ANOVA.

**Figure 2.**
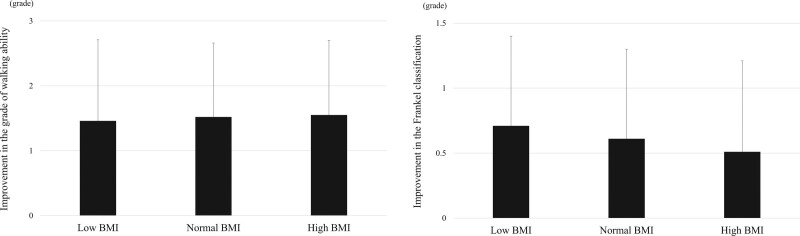
Comparison of the improvement in the grade of walking ability (A) and in Frankel classification (B) among the BMI groups at final follow-up from preoperative baseline. Values are mean ± standard deviation; no statistical significance among the 3 groups was determined after a 1-way ANOVA. BMI = body mass index.

Results of the patients’ pain and ADL measurements are shown in Table 3. The VAS score for LBP and leg pain before surgery and at final follow-up were not significantly different among the groups. Figure 1 shows the improvement in VAS scores at final follow-up from preoperative baseline. There was no significant difference among the groups in improvement of LBP (Fig. 1A). Leg pain showed a significantly smaller improvement in the low BMI group compared with the high BMI group (26.7 vs 42.8 mm, Fig. 1B). Among all patients, the mean preoperative JOA score and score at final follow-up, were 4.5 and 9.9, respectively, and the mean recovery rate was 49.4%, without any significant differences among the 3 groups. Walking ability by grades was not significantly different before surgery or at the final follow-up; although the proportion of patients who could walk independently at the final follow-up was significantly smaller in the low BMI group (75.0%) than in the high BMI (94.3%) groups. The improvement of walking ability and the Frankel classification at final follow-up from preoperative baseline was not statistically different among the groups (Fig. 2).

**Table 3:** Pain and ADL measurements.

	BMI				
	Low, n = 24	Normal, n = 160	High, n = 53	All, n = 237	*P* value
VAS for LBP, mm					
Before surgery	75.5 ± 23.4	72.6 ± 21.8	78.3 ± 21.6	74.1 ± 22.0	.64
At final follow-up	31.5 ± 26.1	29.7 ± 23.8	31.0 ± 21.9	30.2 ± 23.6	.36
VAS for leg pain, mm					
Before surgery	57.0 ± 30.6	54.2 ± 32.0	55.9 ± 29.3	54.9 ± 31.2	.51
At final follow-up	30.2 ± 27.7	19.7 ± 23.0	20.3 ± 19.1	20.8 ± 22.7	.20
JOA score, points					
Before surgery	2.9 ± 3.7	4.5 ± 3.5	5.3 ± 3.6	4.5 ± 3.6	.95
At final follow-up	8.8 ± 3.8	9.7 ± 3.6	10.8 ± 2.7	9.9 ± 3.5	.72
Recovery rate of JOA score, %	44.3 ± 39.7	51.2 ± 28.1	49.3 ± 46.7	49.4 ± 34.2	.13
Walking ability before surgery, n (%)					
Grade 1: unable to walk	6 (25.0)	24 (15.0)	4 (7.5)	34 (14.3)	.51
Grade 2: required caregiver	11 (45.8)	77 (48.1)	27 (50.9)	115 (48.5)	
Grade 3: walker/2 canes	4 (16.7)	22 (13.8)	10 (18.9)	36 (15.2)	
Grade 4: 1 cane	1 (4.2)	28 (17.5)	8 (15.1)	37 (15.6)	
Grade 5: No need for walking aid	2 (8.3)	9 (5.6)	4 (7.5)	15 (6.3)	
Walking ability at final follow-up, n (%)					
Grade 1: unable to walk	1 (4.2)	1 (0.6)	0	2 (0.9)	.26
Grade 2: required caregiver	5 (20.8)	15 (9.5)	3 (5.7)	23 (9.8)	
Grade 3: walker/2 canes	2 (8.3)	30 (19.0)	8 (15.1)	40 (17.0)	
Grade 4: 1 cane	8 (33.3)	46 (29.1)	18 (34.0)	72 (30.6)	
Grade 5: No need for walking aid	8 (33.3)	66 (41.8)	24 (45.3)	98 (41.7)	
Independent walking, n (%)					
Before surgery	7 (29.2)	59 (36.9)	22 (41.5)	88 (37.1)	.58
At final follow-up	18 (75.0) †	142 (89.9)	50 (94.3) †	210 (89.4)	**.04**
Frankel classification before surgery, n (%)					
A	0	1 (0.6)	0	1 (0.4)	.26
B	1 (4.2)	2 (1.3)	1 (1.9)	4 (1.7)	
C	12 (50.0)	72 (45.0)	13 (24.5)	97 (40.9)	
D	10 (41.7)	71 (44.4)	32 (60.4)	113 (47.7)	
E	1 (4.2)	14 (8.8)	7 (13.2)	22 (9.3)	
Frankel classification at final follow-up, n (%)					
A	0	1 (0.6)	1 (1.9)	2 (0.9)	.47
B	1 (4.2)	2 (1.3)	0	3 (1.3)	
C	1 (4.2)	7 (4.4)	0	8 (3.4)	
D	15 (62.5)	101 (63.9)	30 (56.6)	146 (62.1)	
E	7 (29.2)	47 (29.7)	22 (41.5)	76 (32.3)	

Values represent mean ± standard deviation or n (%).

ADL = activities of daily living, JOA = Japanese Orthopaedic Association, LBP = low back pain, VAS = visual analogue scale.

Comparison among 3 groups were performed using 1-way ANOVA for continuous variables and Pearson’s χ^2^ test for categorical variables. A *P* value < 0.05 was statistically significant. post hoc pairwise comparisons between the low and the high BMI groups: †*P* < .05.

### 3.4. Complication-related factors

The overall mean incidence of perioperative complications was 14.3% as shown in Table 4. Mechanical failure occurred in 30.8% of patients on average within the mean follow-up period of 4.0 years. Postoperative new-onset vertebral fractures were noted in an average 32.2% of patients, the reoperation rate was 12.8%, and more than 1 reoperation was performed in 2.1% of patients. None of the factors related to surgical complications were significantly different among the BMI groups.

**Table 4:** Complication-related factors.

	BMI				
	Low, n = 24	Normal, n = 160	High, n = 53	All, n = 237	*P* value
Perioperative complications	4 (16.7)	24 (15.0)	6 (11.3)	34 (14.3)	.76
Mechanical failure	8 (33.3)	50 (31.3)	15 (28.3)	73 (30.8)	.89
Postoperative vertebral fracture	7 (29.2)	52 (32.7)	17 (32.1)	76 (32.2)	.94
Nonunion of fractured vertebra	2 (9.1)	4 (2.6)	3 (5.7)	9 (3.9)	.26
Reoperation	0	23 (14.6)	7 (13.2)	30 (12.8)	.14
Multiple reoperation	0	5 (3.2)	0	5 (2.1)	.21

Values represent mean ± standard deviation or n (%).

BMI = body mass index.

A *P* value < .05 was statistically significant.

## 4. Discussion

This retrospective, multicenter study evaluated OVFs in patients with neurological disorder who underwent fusion surgery. A lower BMI negatively impacted the clinical results despite equivalent radiological results and a complication prevalence, relative to postoperative less improvement in the leg pain and less proportion of patients with independent walking ability.

In this study, preoperative BMD was not significantly different among the low, normal, and the high BMI groups. A lower BMD was associated with not only a lower BMI, but also older age.^[4,5,12]^ In this study, the age of patients in the normal BMI group was significantly older than it was in the high BMI group (75.3 ± 7.9 vs 71.6 ± 7.9), but no significant difference was noted in the low BMI group compared with the normal and the high BMI groups. A relatively younger age in the low BMI group might contribute to the lack of significant difference in BMD compared with the other BMI groups. Despite similar background characteristics among the patients with osteoporosis in this study, the improvement in leg pain was significantly less in the low BMI group compared with the high BMI group (Fig. 1A), and the low BMI group had the lowest proportion of patients who could walk independently at the final follow-up (75.0%); a significant difference existed with the high BMI group (94.3%), but did not exist with the normal BMI group (89.9%). Few reports have identified a low BMI as a risk factor for a poor outcome after spinal surgery. Tarrant et al showed patients with a low BMI who undergo surgery for adolescent idiopathic scoliosis had an increased risk of postoperative ileus.^[13]^ Ottesen et al revealed adverse events to be significantly elevated after anterior cervical spine surgery for patients who are either underweight or super morbidly obese (BMI ≥ 50 kg/m^2^).^[14]^ In another field, a low BMI has also been identified as a risk factor for poor postoperative outcome following vascular surgery, emergency abdominal surgery, and/or shoulder arthroscopy.^[15–17]^ BMI is also a major determinant by which to gauge nutritional status and a low BMI is a risk factor for reduced functional capabilities in the elderly.^[18–20]^ Recently, sarcopenia has been reported to be significantly related to a lower BMI in the Japanese community-dwelling population.^[21]^ Also, a nutritional assessment score is reported to be associated with sarcopenia, as are older age, and/or female gender in Taiwan.^[22]^ Another nationwide cohort study conducted in Canada demonstrated frailty is important predictor of postoperative complications and discharge to a higher level of care in patients undergoing surgery for degenerative spine disease.^[23]^ Factors related to sarcopenia or frailty, which are closely related with poor nutrition, might hamper the recovery of a neurological deficit. On the contrary, a recent retrospective cohort study in which 51,149 patients undergoing cervical spinal surgery were assessed revealed adverse outcomes were less common in overweight/obese patients (BMI: 25.0–49.9 kg/m^2^) compared with patients who had a normal BMI (18.5–24.9 kg/m^2^);^[15]^ this indicates some positive gains can occur in overweight patients, consistent with the results in this study. Patients with the high BMI might be under situation with higher mechanical stress on bone, higher compensation by the body to strengthen the bone and/or hormone levels such as insulin, leptin, and estrogen and anti-inflammatory adipokines, and larger “protective” effect of a fat “cushion” to absorb impact, possibly resulted in the protective effects referred to as *the obesity paradox*.^[24,25]^

There are several limitations of this study. *First, the sample size was relatively small. There are only 24 patients in low BMI group, and this imbalance among groups classified with their BMI might influence the statistical evaluation. Still, this study is nationwide multicenter study, and might have led to less sampling bias.* Second, the high BMI group showed relatively mild obesity (mean BMI: 28.1 kg/m^2^). However, we consider the influence of the mildly obese patients is limited in this study given that the percentage of body fat is generally higher among Asian people. As the World Health Organization suggest, the risk of obesity-related diseases among Asian people rises starting at a BMI of 23 kg/m^2^ compared with from 25 kg/m^2^ for non-Asian people.^[26]^ *Third,* this was a retrospective, multicenter, study based on a review of medical records. The treatment for OVF, indications for fusion surgery, detailed surgical methods and concepts, fusion levels or indications for osteotomy, and postoperative course (e.g., rehabilitation, orthoses usage, etcetera) may vary by institute. These factors could influence the radiological or clinical results. *Forth*, we evaluated ADL or quality of life using the JOA score for lumbar disease at the thoracic and thoracolumbar levels. The JOA score is a comprehensive outcome that was originally used for lumbar disease with low back pain consisting of objective and subjective findings, as well as assessments of bladder and bowel dysfunction. This score is not yet validated for the assessment of extensive back pain in thoracic vertebral fractures. Further investigation is needed into the impact of BMI on clinical outcomes after OVF surgery to reveal the underlying mechanism linking BMI and its effect on neural recovery.

## 5. Conclusions

This study was designed to provide the first clinical and radiological data for the evaluation of fusion surgery that focused on the BMI spectrum in a large sample of patients with OVFs. The study identified patients with a low BMI who were representative of an at-risk population for leg pain and a poor recovery in terms of independent walking ability after surgery. Alternatively, patients with a high BMI showed some positive improvements in terms of ADL after surgery; although comprehensive data from the higher end of the BMI spectrum are still lacking. Surgeons should recognize the importance of BMI as a factor that can affect clinical outcomes when fusion surgery for OVF in older patients is indicated.

## Author contributions

**Conceptualization:** Gen Inoue, Masayuki Miyagi, Wataru Saito, Eiki Shirasawa, Kentaro Uchida, Masashi Takaso.

**Data curation:** Gen Inoue, Masayuki Miyagi, Wataru Saito, Eiki Shirasawa, Kentaro Uchida, Naobumi Hosogane, Kei Watanabe, Keiichi Katsumi, Takashi Kaito, Tomoya Yamashita, Hiroyasu Fujiwara, Yukitaka Nagamoto, Kenya Nojiri, Satoshi Suzuki, Eijiro Okada, Seiji Ueda, Tomohiro Hikata, Yuta Shiono, Kota Watanabe, Hidetomi Terai, Koji Tamai, Yuji Matsuoka, Hidekazu Suzuki, Hirosuke Nishimura, Atsushi Tagami, Syuta Yamada, Shinji Adachi, Seiji Ohtori, Takeo Furuya, Sumihisa Orita, Kazuhide Inage, Toshitaka Yoshii, Shuta Ushio, Haruki Funao, Norihiro Isogai, Katsumi Harimaya, Seiji Okada, Kenichi Kawaguchi, Nobuhiko Yokoyama, Hidekazu Oishi, Toshiro Doi, Katsuhito Kiyasu, Shiro Imagama, Kei Ando, Kazuyoshi Kobayashi, Daisuke Sakai, Masahiro Tanaka, Atsushi Kimura, Hirokazu Inoue, Atsushi Nakano, Shota Ikegami, Masayuki Shimizu, Toshimasa Futatsugi, Kenichiro Kakutani, Takashi Yurube, Kazuyoshi Nakanishi, Masashi Oshima, Hiroshi Uei, Yasuchika Aoki, Masahiko Takahata, Akira Iwata, Hirooki Endo, Shoji Seki, Hideki Murakami, Satoshi Kato, Katsuhito Yoshioka, Michio Hongo, Tetsuya Abe, Toshinori Tsukanishi, Masashi Takaso, Ken Ishii.

**Formal analysis:** Gen Inoue, Masayuki Miyagi.

**Investigation:** Gen Inoue, Masayuki Miyagi, Wataru Saito, Eiki Shirasawa, Kentaro Uchida, Naobumi Hosogane, Kei Watanabe, Keiichi Katsumi, Takashi Kaito, Tomoya Yamashita, Hiroyasu Fujiwara, Yukitaka Nagamoto, Kenya Nojiri, Satoshi Suzuki, Eijiro Okada, Seiji Ueda, Tomohiro Hikata, Yuta Shiono, Kota Watanabe, Hidetomi Terai, Koji Tamai, Yuji Matsuoka, Hidekazu Suzuki, Hirosuke Nishimura, Atsushi Tagami, Syuta Yamada, Shinji Adachi, Seiji Ohtori, Takeo Furuya, Sumihisa Orita, Kazuhide Inage, Toshitaka Yoshii, Shuta Ushio, Haruki Funao, Norihiro Isogai, Katsumi Harimaya, Seiji Okada, Kenichi Kawaguchi, Nobuhiko Yokoyama, Hidekazu Oishi, Toshiro Doi, Katsuhito Kiyasu, Shiro Imagama, Kei Ando, Kazuyoshi Kobayashi, Daisuke Sakai, Masahiro Tanaka, Atsushi Kimura, Hirokazu Inoue, Atsushi Nakano, Shota Ikegami, Masayuki Shimizu, Toshimasa Futatsugi, Kenichiro Kakutani, Takashi Yurube, Kazuyoshi Nakanishi, Masashi Oshima, Hiroshi Uei, Yasuchika Aoki, Masahiko Takahata, Akira Iwata, Hirooki Endo, Shoji Seki, Hideki Murakami, Satoshi Kato, Katsuhito Yoshioka, Michio Hongo, Tetsuya Abe, Toshinori Tsukanishi, Masashi Takaso, Ken Ishii.

**Methodology:** Kentaro Uchida, Naobumi Hosogane.

**Project administration:** Ken Ishii

**Supervision:** Naobumi Hosogane, Takashi Kaito, Kota Watanabe, Seiji Ohtori, Katsumi Harimaya, Seiji Okada, Shiro Imagama, Kazuyoshi Nakanishi, Yasuchika Aoki, Hideki Murakami, Satoshi Kato, Masashi Takaso, Ken Ishii.

**Writing – original draft:** Gen Inoue.

**Writing – review & editing:** Masayuki Miyagi, Wataru Saito, Eiki Shirasawa, Kentaro Uchida, Masashi Takaso.
